# CD226 identifies functional CD8^+^T cells in the tumor microenvironment and predicts a better outcome for human gastric cancer

**DOI:** 10.3389/fimmu.2023.1150803

**Published:** 2023-03-28

**Authors:** Hao Huang, Ziyi Huang, Junwei Ge, Jiayi Yang, Junjun Chen, Bin Xu, Shaoxian Wu, Xiao Zheng, Lujun Chen, Xueguang Zhang, Jingting Jiang

**Affiliations:** ^1^ Department of Tumor Biological Treatment, The Third Affiliated Hospital of Soochow University, Changzhou, Jiangsu, China; ^2^ Jiangsu Engineering Research Center for Tumor Immunotherapy, The Third Affiliated Hospital of Soochow University, Changzhou, Jiangsu, China; ^3^ Institute of Cell Therapy, The Third Affiliated Hospital of Soochow University, Changzhou, Jiangsu, China; ^4^ Jiangsu Institute of Clinical Immunology, The First Affiliated Hospital of Soochow University, Suzhou, Jiangsu, China; ^5^ Jiangsu Key Laboratory of Clinical Immunology, Soochow University, Suzhou, Jiangsu, China; ^6^ Jiangsu Key Laboratory of Gastrointestinal Tumor Immunology, Soochow University, Suzhou, Jiangsu, China

**Keywords:** CD226, CD8^+^ T cells, multi-color immunohistochemical staining, prognosis, gastric cancer

## Abstract

It is well-known that CD226 serves as a critical activating receptor on various immune cells, such as lymphocytes and monocytes, and it is suggested to promote anti-tumor immunity in the tumor microenvironment (TME). Herein, we showed a crucial regulatory role of CD226 in CD8^+^T cell-mediated anti-tumor response in TME of human gastric cancer (GC). Specifically, the increased CD226 expression in cancer tissues was significantly associated with better clinical outcomes in GC patients. Moreover, the increased infiltrating CD226^+^CD8^+^T cells and the increased ratio of infiltrating CD226^+^CD8^+^T cells in CD8^+^T subpopulation within cancer tissues could also be valuable prognostic predictors for GC patients. Mechanically, the assay for transposase-accessible chromatin using sequencing (ATAC-seq) analysis revealed that the chromatin accessibility of *CD226* in CD4^+^ and CD8^+^TILs was significantly higher than that in CD8^+^T cells in normal tissues. Further analysis showed that CD8^+^TILs highly expressed immune checkpoint molecules, such as *TIGIT*, *LAG3*, and *HAVCR2*, which means CD8^+^TILs are more exhausted. In addition, our multi-color immunohistochemical staining (mIHC) revealed that GC patients with higher frequency of IFN-γ^+^CD226^+^CD8^+^TILs showed poorer prognosis. Combined with the single-cell transcriptome sequencing (scRNA-seq) data analysis, we found that the expressions of IFN-γ and TIGIT in CD8^+^TILs were significantly and positively correlated. The expression of TIGIT in IFN-γ^+^CD226^+^CD8^+^TILs was higher, while that in IFN-γ^-^CD226^+^CD8^+^TILs was significantly lower. The correlation analysis showed that the expression of *CD226* was positively correlated with the score of effector T cells but negatively correlated with that of immunosuppressive factors, such as Tregs and tumor-associated macrophages (TAMs). Collectively, we showed that the frequency of CD226^+^CD8^+^TILs was an excellent prognostic predictor for GC patients. Our findings provided insights into the interaction pattern between co-stimulatory receptor CD226 and tumor cells as well as the infiltrating immune cells in the TME in GC.

## Introduction

Cancer immunotherapy has been considered as an effective and vital adjuvant strategy for traditional surgery, chemotherapy, radiotherapy, and targeted therapy and even has been selected as the first-line treatment against cancer in clinical practice (1). As the most frequently diagnosed digestive tract malignancy, gastric cancer (GC) represents one of the most common causes of cancer death worldwide. Although a recent decline in the mortality of GC has been found in rural and urban areas in China, the clinical outcomes of GC patients in the advanced stage still remain poor (2, 3). Therefore, the personalized therapeutic strategy based on the classification of molecular biomarkers, such as microsatellite instability (MSI), programmed cell death ligand 1 (PD-L1), and human epidermal growth factor receptor 2 (HER2), would help the patients benefit from immunotherapy and targeted therapy (3). Moreover, different subtypes and various abundances of infiltrating immune cells in GC tissues can also be used to evaluate the response to immunotherapy and to predict the patients’ outcomes (3–5). In addition, among these immune cells, the CD8^+^T cells are considered as the essential and preferred subsets for anti-tumor immunity, and the numbers and the effector functions of these cells contribute essentially to determining the outcome of GC patients (6). We have also previously reported that the type I lymphocytes, such as T-bet^+^CD8^+^T cells, are increased in GC tissues compared with adjacent normal tissues, showing improved survival of GC patients (7).

However, despite the presence of a certain intensity of tumor-infiltrating CD8^+^T cells (CD8^+^TILs) in the tumor microenvironment (TME), it cannot effectively control the tumor progression since the CD8^+^T cells always encounter dysfunction and exhaustion, leading to immunosuppression and tolerance in the TME (8). Therefore, the status of CD8^+^TILs, generally characterized using transcription factors, activating receptors, and inhibitory receptors, plays a vital role in the outcome of anti-tumor immunity (9). Upon activation, co-stimulatory molecules fine-tune CD8^+^T cell response by binding to the surface receptors of lymphocytes and are related to the secretion of interferon-γ (IFN-γ), tumor necrosis factor-α (TNF-α), and granzymes (8). However, the inhibitory receptors, such as PD-1, CTLA-4, TIM-3, LAG3, TIGIT, and CD39, mediate the suppressive effects to terminate immune responses and lead to immune escape (10–12). Since the CD8^+^TILs have high heterogeneity and most CD8^+^TILs are insensitive to tumors, CD8^+^TILs are also defined as bystander T cells. Therefore, it is necessary to further investigate whether these bystander CD8^+^TILs express specific markers associated with immune activation, stimulation, or inhibition.

CD226, also known as DNAM1 (DNAX accessory molecule-1), is broadly expressed on T cells, natural killer (NK) cells, platelets, monocytes, and a subset of B cells, and it is also identified as a crucial co-activating receptor to restrain CD8^+^T-mediated anti-tumor response (13). CD226 consists of two extracellular immunoglobulin-like domains and an intracellular immunoglobulin tail tyrosine-like motif (ITT), and it delivers co-stimulatory signals through the ITT/ITT-like motif (14, 15). CD226, TIGIT, and CD96 share the same ligand CD155 (also known as PVR/NECL5/TAGE4), a member of the nectin-like family of adhesion molecules and highly expressed on cancer cells (16). Unlike CD226, an activated receptor that delivers a positive stimulatory signal to the immune cells, TIGIT and CD96 are well-known inhibitory checkpoint receptors and participate in the immune suppression of TME (16). He et al. have shown that the peripheral CD8^+^T cells expressed a higher level of TIGIT in GC patients compared with healthy controls, while a lower level of peripheral CD226^+^CD8^+^T cells has been found in GC patients compared with healthy controls (17). Moreover, they have also confirmed that TIGIT conjugated with CD155 on GC cells significantly alters the metabolic reprogramming of CD8^+^T cells and promotes cancer progression (17). Jin et al. have shown that CD226^low^CD8^+^TILs have an exhausted phenotype with impaired functionality, while CD226^high^CD8^+^TILs have greater self-renewal capacity and responsiveness, and the higher intensity of CD226^high^CD8^+^TILs may improve responses to anti-TIGIT therapy (18).

In our present study, we aimed to examine the clinical associations and prognostic values of CD226^+^CD8^+^TILs in human GC tissues. Moreover, we investigated the regulatory role of CD226 in the effector function of CD8^+^TILs by analyzing published single-cell RNA sequencing (scRNA-seq) data and bulk assay for ATAC-seq data of TILs in MC38 model downloaded from the Gene Expression Omnibus (GEO) dataset. We showed that the intensity of CD226^+^CD8^+^TILs could be a useful prognostic predictor for GC patients. Furthermore, we provided insights into the interplay between co-stimulatory receptor CD226 and tumor cells as well as other infiltrating immune cells in the TME of GC.

## Materials and methods

### Patients and tissue specimens

The human GC tissue microarray (TMA, catalog: HStmA180Su08) was provided by Shanghai Outdo Biotech Co., Ltd., Shanghai, China. A total of 98 patients (64 males and 34 females, aged 32 to 81 years) who underwent surgery from July 2006 to April 2007 were enrolled in this study. The detailed clinical parameters of these patients are shown in **Tables 1**-**5**.

**Table 1:** The association between the percentages of CD8^+^T cells, CD8^+^T cells in the epithelial cell region, CD8^+^T cells in the stromal cell region in tumor and clinical features of GC patients.

Clinical parameters	cases	CD8^+^T cells infiltration		*OR*	*χ2*	*P*-value	CD8^+^T cells infiltration		*OR*	*χ2*	*P*-value	CD8^+^T cells infiltration		*OR*	*χ2*	*P*-value
		Low	High				Low	High				Low	High			
Gender																
Male	64	35	29	1.207	0.196	0.658	39	25	1.092	0.041	0.839	33	31	1.198	0.180	0.671
Female	34	17	17				20	14				16	18			
Age (years)																
<66	50	26	24	0.910	0.053	0.818	29	21	0.888	0.082	0.775	26	24	1.182	0.167	0.683
≥66	46	25	21				28	18				22	24			
Tumor size (cm)																
<5.5	48	20	28	0.392	5.061	**0.025**	23	25	0.379	5.225	**0.022**	19	29	0.429	4.167	**0.041**
≥5.5	48	31	17				34	14				29	19			
T stage																
T_1-2 _	15	5	10	0.391	2.635	0.105	7	8	0.532	1.272	0.259	6	9	0.635	0.639	0.424
T_3-4_	82	46	36				51	31				42	40			
N stage																
N_0_	27	11	16	0.486	2.491	0.115	13	14	0.484	2.523	0.112	11	16	0.579	1.430	0.232
N_1-3_	70	41	29				46	24				38	32			
M stage																
M_0_	89	46	43	0.357	0.804	0.370	51	38	0	–	**0.021**	43	46	0.312	1.160	0.282
M_1_	8	6	2				8	0				6	2			
Pathological stage																
II+III	87	45	42	0.612	0.181	0.671	52	35	0.849	0.006	0.936	43	44	0.814	0.102	0.749
IV	11	7	4				7	4				6	5			
TNM stage																
I+II	41	20	21	0.685	0.002	0.964	22	19	0.563	1.838	0.175	21	20	1.013	0.0009	0.976
III+IV	55	32	23				37	18				28	27			

The values of cutoff point were 4.347 (in total region), 0.038 (in epithelial region), 0.067 (in stromal region). Values higher than the cutoff point were defined as “High”, and others were defined as “Low”. Bold signifies P<0.05.

**Table 2:** The association between the percentages of CD226^+^ cells, CD226^+^ cells in the epithelial cell region, and CD226^+^ cells in the stromal cell region in tumor and clinical features of GC patients.

Clinical parameters	cases	CD226^+^ cells infiltration		*OR*	*χ* ^2^	*P-*value	CD226^+^ cells infiltration		*OR*	*χ* ^2^	*P-*value	CD226^+^ cells infiltration		*OR*	*χ* ^2^	*P-*value
		Low	High				Low	High				Low	High			
Gender																
Male	64	37	27	1.542	1.034	0.309	48	16	1.435	0.601	0.438	46	18	1.065	0.018	0.893
Female	34	16	18				23	11				24	10			
Age (years)																
<66	50	23	27	0.499	2.803	0.094	32	18	0.432	3.201	0.074	30	20	0.316	5.928	**0.015**
≥66	46	29	17				37	9				38	8			
Tumor size (cm)																
<5.5	48	23	25	0.603	1.510	0.219	34	14	0.810	0.211	0.646	31	17	0.480	2.525	0.112
≥5.5	48	29	19				36	12				38	10			
T stage																
T_1-2_	15	7	8	0.719	0.344	0.558	10	5	0.733	0.267	0.605	11	4	1.138	0.011	0.916
T_3-4_	82	45	37				60	22				58	24			
N stage																
N_0_	27	15	12	1.053	0.013	0.910	18	9	0.642	0.813	0.367	20	7	1.143	0.068	0.794
N_1-3_	70	38	32				53	17				50	20			
M stage																
M_0_	89	46	43	0.153	2.491	0.115	64	25	0.366	0.288	0.591	62	27	0.328	0.435	0.510
M_1_	8	7	1				7	1				7	1			
Pathological stage																
II+III	87	48	39	1.477	0.371	0.542	62	25	0.551	0.144	0.704	62	25	0.930	0.064	0.800
IV	11	5	6				9	2				8	3			
TNM																
I+II	41	22	19	0.896	0.070	0.792	28	13	0.601	1.193	0.275	32	9	1.730	1.349	0.245
III+IV	55	31	24				43	12				37	18			

The values of cutoff point were 3.683 (in total region), 0.053 (in epithelial region), 0.070 (in stromal region). Values higher than the cutoff point were defined as “High”, and others were defined as “Low”. Bold signifies P<0.05.

**Table 3:** The association between the percentages of CD8^+^CD226^+^T cells, CD8^+^CD226^+^T cells in the epithelial cell region, and CD8^+^CD226^+^T cells in the stromal cell region in tumor and clinical features of GC patients.

Clinical parameters	cases	CD8^+^CD226^+^T cells infiltration		*OR*	*χ* ^2^	*P-*value	CD8^+^CD226^+^T cells infiltration		*OR*	*χ* ^2^	*P-*value	CD8^+^CD226^+^T cells infiltration		*OR*	*χ* ^2^	*P-*value
		Low	High				Low	High				Low	High			
Gender																
Male	64	28	36	1.426	0.657	0.418	43	21	0.979	0.002	0.963	33	31	0.946	0.017	0.897
Female	34	12	22				23	11				18	16			
Age (years)																
<66	50	15	35	0.393	4.884	**0.027**	29	21	0.434	3.527	0.060	20	30	0.391	5.091	**0.024**
≥66	46	24	22				35	11				29	17			
Tumor size (cm)																
<5.5	48	14	34	0.379	5.225	**0.022**	28	20	0.416	3.859	**0.0495**	20	28	0.429	4.174	**0.041**
≥5.5	48	25	23				37	11				30	18			
T stage																
T_1-2_	15	3	12	0.319	2.101	0.147	7	8	0.362	3.322	0.068	5	10	0.411	2.357	0.125
T_3-4 _	82	36	46				58	24				45	37			
N stage																
N_0_	27	8	19	0.500	2.080	0.149	17	10	0.729	0.444	0.505	12	15	0.636	0.993	0.319
N_1-3_	70	32	38				49	21				39	31			
M stage																
M_0_	89	34	55	0.206	2.724	0.099	57	32	0	–	**0.0496**	45	44	0.341	0.915	0.339
M_1_	8	6	2				8	0				6	2			
Pathological stage																
II+III	87	36	51	1.235	0.102	0.750	57	30	0.422	0.555	0.456	46	41	1.346	0.215	0.643
IV	11	4	7				9	2				5	6			
TNM stage																
I+II	41	13	28	0.481	2.920	0.088	26	15	0.711	0.603	0.437	20	21	0.737	0.542	0.461
III+IV	55	27	28				39	16				31	24			

The values of cutoff point were 0.00084 (in total region), 0.004 (in epithelial region), 0.004 (in stromal region). Values higher than the cutoff point were defined as “High”, and others were defined as “Low”. Bold signifies P<0.05.

**Table 4:** The association between the percentages of CD8^+^CD226^+^T cells/CD8^+^T cells, CD8^+^CD226^+^T cells/CD8^+^T cells in the epithelial cell region, and CD8^+^CD226^+^T cells/CD8^+^T cells in the stromal cell region in tumor and clinical features of GC patients.

Clinical parameters	cases	CD8^+^CD226^+^T cellsamong CD8^+^T cells		*OR*	*χ* ^2^	*P-*value	CD8^+^CD226^+^T cellsamong CD8^+^T cells		*OR*	*χ* ^2^	*P-*value	CD8^+^CD226^+^T cellsamong CD8^+^T cells		*OR*	*χ* ^2^	*P-*value
		Low	High				Low	High				Low	High			
Gender																
Male	64	28	36	1.426	0.657	0.418	51	13	0.523	0.614	0.433	38	26	1.299	0.375	0.540
Female	34	12	22				30	4				18	16			
Age (years)																
<66	50	13	37	0.270	9.253	**0.002**	38	12	0.386	2.835	0.092	22	28	0.310	7.534	**0.006**
≥66	46	26	20				41	5				33	13			
Tumor size (cm)																
<5.5	48	14	34	0.379	5.225	**0.022**	38	10	0.543	1.200	0.273	22	26	0.385	5.151	**0.023**
≥5.5	48	25	23				42	6				33	15			
T stage																
T_1-2_	15	3	12	0.319	2.101	0.147	9	6	0.232	6.200	**0.013**	5	10	0.320	3.947	**0.047**
T_3-4_	82	36	46				71	11				50	32			
N stage																
N_0_	27	11	16	0.972	0.004	0.951	23	4	1.190	0.0008	0.977	13	14	0.583	1.408	0.235
N_1-3_	70	29	41				58	12				43	27			
M stage																
M_0_	89	34	55	0.206	2.724	0.099	72	17	0	–	0.344	49	40	0.408	0.516	0.473
M_1_	8	6	2				8	0				6	2			
Pathological stage																
II+III	87	36	51	1.235	0.000	0.995	71	16	0.444	0.119	0.730	50	37	1.126	0.034	0.853
IV	11	4	7				10	1				6	5			
TNM stage																
I+II	41	16	25	0.827	0.206	0.650	33	8	0.702	0.417	0.518	21	20	0.649	1.078	0.299
III+IV	55	24	31				47	8				34	21			

The values of cutoff point were 0.00036 (in total region), 0.133 (in epithelial region), 0.068 (in stromal region). Values higher than the cutoff point were defined as “High”, and others were defined as “Low”. Bold signifies P<0.05.

**Table 5:** The association between the percentages of CD226^+^CD8^+^IFN-γ^+^T cells/CD8^+^CD226^+^T cells, CD226^+^CD8^+^IFN-γ^+^T cells/CD8^+^CD226^+^T cells in the epithelial cell region, and CD226^+^CD8^+^IFN-γ^+^T cells/CD8^+^CD226^+^T cells in the stromal cell region in tumor and clinical features of GC patients.

Clinical parameters	cases	CD8^+^CD226^+^IFN-γ^+^T cells among CD8^+^CD226^+^T cells		*OR*	*χ* ^2^	*P-*value	CD8^+^CD226^+^IFN-γ^+^T cells among CD8^+^CD226^+^T cells		*OR*	*χ* ^2^	*P-*value	CD8^+^CD226^+^IFN-γ^+^T cells among CD8^+^CD226^+^T cells		*OR*	*χ* ^2^	*P-*value
		Low	High				Low	High				Low	High			
Gender																
Male	64	49	15	1.005	0.0001	0.992	60	4	2.000	0.315	0.574	58	6	0.935	0.077	0.781
Female	34	26	8				30	4				31	3			
Age (years)																
<66	50	36	14	0.541	1.526	0.217	46	4	0.802	0.013	0.909	45	5	0.857	0.017	0.895
≥66	46	38	8				43	3				42	4			
Tumor size (cm)																
<5.5	48	38	10	1.411	0.515	0.473	46	2	3.286	1.227	0.268	46	2	3.927	1.962	0.161
≥5.5	48	35	13				42	6				41	7			
T stage																
T_1-2_	15	12	3	1.290	0.001	0.970	14	1	1.307	0.072	0.788	14	1	1.514	0.011	0.917
T_3-4_	82	62	20				75	7				74	8			
N stage																
N_0_	27	24	3	2.769	1.664	0.197	26	1	2.889	0.358	0.550	26	1	3.355	0.616	0.433
N_1-3_	70	52	18				63	7				62	8			
M stage																
M_0_	89	68	21	0.463	0.077	0.782	82	7	1.673	0.046	0.830	81	8	1.446	0.095	0.758
M_1_	8	7	1				7	1				7	1			
Pathological stage																
II+III	87	70	17	3.431	3.767	0.052	81	6	3.000	0.495	0.482	82	5	9.371	7.611	**0.006**
IV	11	6	5				9	2				7	4			
TNM stage																
I+II	41	35	6	2.188	2.196	0.138	40	1	5.833	2.047	0.153	40	1	6.809	2.753	0.097
III+IV	55	40	15				48	7				47	8			

The values of cutoff point were 0 (in total region), 0.372 (in epithelial region), 0 (in stromal region). Values higher than the cutoff point were defined as “High”, and others were defined as “Low”. Bold signifies P<0.05.

### Multi-color immunohistochemical staining

The mIHC was carried out by using the Opal 5-color fluorescent IHC kit (catalog No. NEL811001KT, PerkinElmer, USA) in combination with automated quantitative analyses (PerkinElmer, USA) based on the manufacturer’s instructions to detect three lymphocyte markers CD226, CD8, IFN-γ, and cytokeratin (CK) in tumor tissues. CK was used to identify the malignant epithelial cells, and 4’,6-diamidino-2-phenylindole (DAPI) was used to stain the nucleus. Briefly, the concentrations of the four antibodies were optimized, and the spectral library was established based on the single-stained slides. Deparaffinization, rehydration, and antigen retrieval of the human GC TMA were deparaffinized with xylenes, rehydrated through graded alcohols, and rinsed with ddH_2_O and 1X PBS following standard protocols. Heat-induced epitope retrieval was performed in EDTA solution pH=9.0 in a pressure cooker for 5 min. The tissue sections were cooled down on the bench top for 1 h. Each section was soaked in 100-200 µL blocking solution at room temperature for 15 min. The primary antibodies used were as follows, anti-CD226 (1:500 dilution, catalog No. ab214327, Abcam, Cambridge), anti-CD8 (catalog No. PA067, BioDot, USA), anti-IFN-γ (1:500 dilution, catalog No. M0876, DAKO, Denmark), and anti-CK (1:2 dilution, catalog No. PA125, BioDot, USA). The GC TMA slide was then incubated with HRP-conjugated secondary-antibodies (PerkinElmer, USA) in Opal working solution (PerkinElmer, USA). The slide was mounted with ProLong Diamond Antifade Reagent with DAPI (Thermofisher, USA).

### Integration of multiple scRNA-seq data, dimension reduction, and unsupervised clustering

Processed pan-cancer scRNA-seq data were downloaded from GEO datasets (GSE156728). T cells from various cancers were merged into one Seurat object (19). Genes associated with cell cycle phase score were regressed out by ScaleData function. The batch effect was removed by R package harmony (20). CD4^+^T cells, CD8^+^T cells, and regulatory T (Treg) cells were defined by *CD4*, *CD8A*, and *FOXP3*, respectively. Three CD8^+^TILs groups (CD226^+^CD8^+^TILs, TIGIT^+^CD8^+^TILs, and CD226^+^TIGIT^+^CD8^+^TILs) were identified by *CD226* and *TIGIT*. All visualizations were constructed by Seurat.

### The cancer genome atlas STAD data analysis

TCGA STAD was downloaded from the UCSC Xena website (http://xena.ucsc.edu/). Tumor purity, stromal score, and immune score were estimated by R package estimate. R package pheatmap was used to visualize the correlation between *CD226* and tumor purity, stromal score, and immune score, as well as with cell-specific gene signatures and molecules associated with effector T lymphocytes in TME. CIBERSORT package was applied to calculate the score of 22 cellular components using the file LM22.txt. Dot plot was used to visualize the relationship between *CD226* and the score of cellular components.

### Trajectory analysis

Single-cell trajectory analysis with Monocle2 identified genes using the following criteria: expressed in more than 10 subsets of cells and the average expression > 0.3. The method of ‘DDRTree’ was applied to estimate psedotime. Plot_cell_trajectory function was used to visualize the trajectory.

### Imaging analysis

First, the Tissue FAXS system (Tissue Gnostics Asia Pacific Limited, Austria) was used to conduct panoramic multispectral scanning of the slide, and then acquired images were processed using Strata Quest analysis software (Version No. 7.0.1.165, Tissue Gnostics Asia Pacific Limited, Austria), in which each fluorophore was spectrally unmixed into individual channels and saved as a separate file. DAPI was used to generate a binary mask of all viable cells in the image. Similarly, the expressions of CD8, CD226, and IFN-γ were combined with DAPI to create binary masks of all cells expressing these biomarkers of interest. Finally, the binary mask of CK was counted to obtain local tumor cells (Detailed descriptions are listed in **Tables 1**–**3**). For tissue split, we used pan-CK to divide the tissue area, and the continuous area of CK positive identified by TissueGnostics multiplex imaging technology was divided into the continuous area of CK negative in the tumor parenchyma for the interstitial region.

### Survival analysis

The fluorescence intensity of each protein across different patients was cut off by surv_cutpoint function of R package survminer, and patients were divided into two groups according to cut off value. Then, R package survival was applied to plot survival curve. Uni-variate and multi-variate analysis of Cox model was used coxph function.

### Statistical analysis

Statistical analysis was performed using Prism 7 software (GraphPad) and RStudio 3.6.3. The Chi-square test was used to compare the disease-related factors in patients with low and high expressions of CD8, CD226, and IFN-γ in different cell populations. Log-rank survival analysis was used to predict the postoperative overall survival (OS) of the patients. Cox regression analyses were carried out to determine the expressions of prognostic factors (CD226, CD8, and IFN-γ) for GC. *P*<0.05 was considered statistically significant.

## Results

### CD226 is up-regulated in effector CD4^+^ and CD8^+^T cells

**Figure 1:**
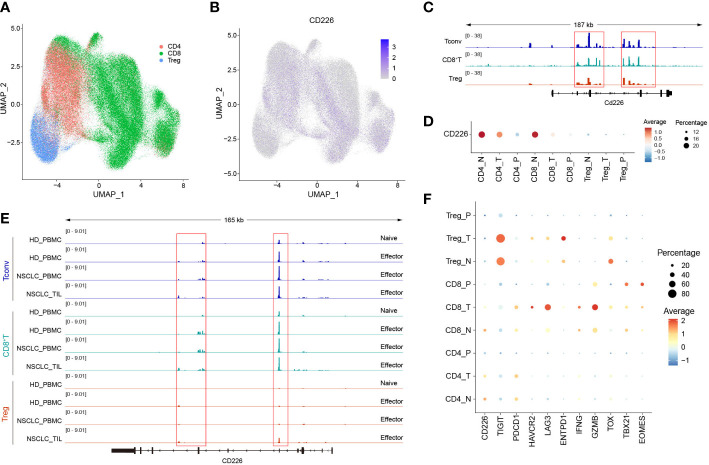
The expression of CD226 is up-regulated in effector CD4^+^ and CD8^+^T cells. **(A, B)** scRNA-seq data downloaded from the GEO dataset (GSE156728), UMAP plots showed the distribution of CD4^+^T cells, CD8^+^T cells, and Tregs and *CD226* expression. **(C)** Bulk ATAC-seq data for TILs in the MC38 model downloaded from the GEO dataset (GSE211155), genome tracks of bulk ATAC-seq data in the *Cd226* locus, grouped by different clusters. **(D)** Dot plot showed the expression of CD226 in different sub-types of T cells from various tissues. **(E)** Genome tracks of bulk ATAC-seq data in *CD226* locus, grouped by different samples. Bulk ATAC-seq data of T cells for healthy donors and non-small cell lung cancer were downloaded from the GEO dataset (GSE211155). **(F)** Dot plot showed the expressions of selected genes in different types of T cells from various tissues.

We downloaded multi-omics sequencing data from public databases and analyzed the expression of CD226 in different tissues and cell populations. First, we collected the single-cell RNA-sequencing pan-cancer atlas of T cells and analyzed the expression of CD226 in CD4^+^T cells, CD8^+^T cells, and Tregs (**Figure 1A**) (21). CD226 was mainly expressed in effector CD4^+^T cells and CD8^+^T cells but was rarely expressed in Tregs (**Figure 1B**). *CD226* locus of CD4^+^ and CD8^+^TILs was also analyzed (22). Consistent with the results of scRNA-seq, the chromatin accessibility of *CD226* in CD4^+^ and CD8^+^TILs was significantly higher compared with infiltrating Tregs (**Figure 1C**). Next, we further explored the expression of *CD226* in different tissues and found that T cells in peripheral blood mononuclear cells (PBMCs) hardly expressed *CD226* (**Figure 1D**), which was also consistent with the results of human bulk ATAC data (**Figure 1E**) (22). Generally, the expression of *CD226* in CD4^+^, CD8^+^T cells, or Tregs in normal tissues was higher than that in tumor tissues (**Figures 1D, E**). As CD226 and TIGIT shared the same ligand, *CD226* was down-regulated, but the inhibitory molecule *TIGIT* was up-regulated in tumors (**Figure 1F**). In addition, the expressions of T cell exhaustion genes, such as *PDCD1*, *LAG3*, *HAVCR2*, and *ENTPD1*, cytotoxic molecules (*IFNG* and *GZMB*), and exhaustion-related transcription factors (*TOX*, *TBX21*, and *EOMES*) were increased in CD8^+^TILs compared with CD4^+^T cells or Tregs (**Figure 1F**).

### Expression and localization of CD8 and CD226 in GC tissues and normal gastric tissues

**Figure 2:**
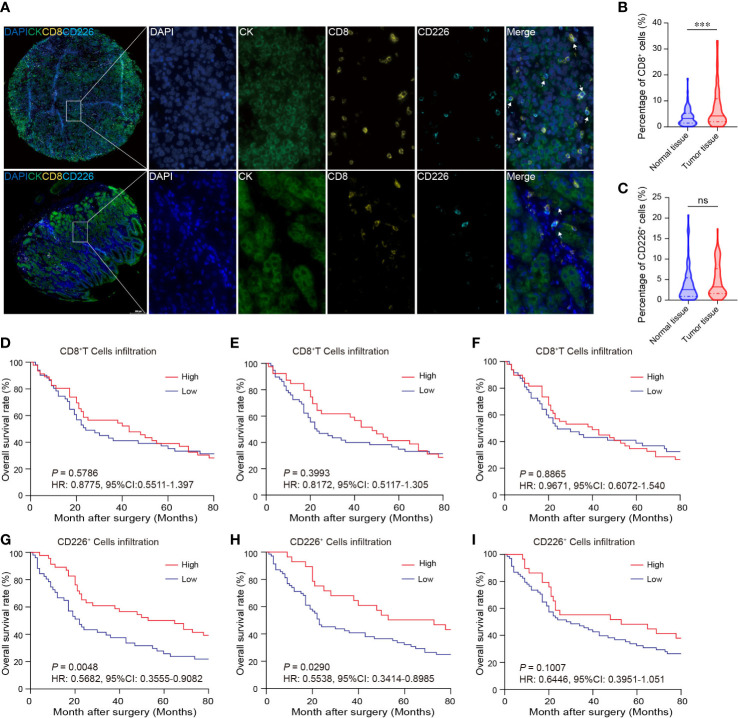
The expressions of CD8 and CD226 in human GC TMA. **(A)**. mIHC and single-color images were obtained from human GC TMA. **(B, C)**. The populations of CD8^+^TILs and CD226^+^TILs were compared between normal tissue and GC tissues. **(D, G)**. Prognostic values of CD8^+^TILs and CD226^+^TILs in GC. **(E, H)**. Prognostic values of CD8^+^TILs and CD226^+^TILs in GC in the epithelial cell region. **(F, I)**. Prognostic values of CD8^+^TILs and CD226^+^TILs in GC in the stromal cell region.

GC tissues and normal gastric tissues were stained by mIHC to reveal the spatial distribution of CD8^+^T cells, CD226^+^ cells, and CK^+^ epithelial cells (**Figure 2A**: CK (green), CD8 (yellow), and CD226 (indigo)). A higher frequency of CD8^+^TILs was observed in GC tissues compared with normal tissues (**Figure 2B**, *P*<0.001). However, we did not find any significant difference in the expression of CD226 between GC tissues and normal tissues (**Figure 2C**).

Tumor-infiltrating cytotoxic CD8^+^T cells can potently suppress tumor growth. Studies have shown that the frequency of CD8^+^TILs can predict the outcomes of patients. Herein, we also investigated the association between the frequency of CD8^+^TILs and patients’ survival in GC. We found no significant association between the frequency of CD8^+^TILs in GC tissues and overall survival (OS) (**Figure 2D**: total CD8^+^TILs, **Figure 2E**: CD8^+^TILs in the epithelial cell region, **Figure 2F**: CD8^+^TILs in the stromal cell region). However, higher frequency of CD226^+^TILs was significantly associated with better clinical outcomes (*HR*=0.5682, 95% CI: 0.3555-0.9082, *P*=0.0048, **Figure 2G**). We also detected CD226^+^TILs in the epithelial cell region and stromal cell region. We found that the patients with higher frequency of CD226^+^TILs in the epithelial cell region had better prognosis (*HR*=0.5538, 95% CI: 0.3414-0.8985, *P*=0.0290, **Figure 2H**), and the prognosis of the patients with higher frequency of CD226^+^TILs in the stromal cell region also tended to be better than those with higher frequency of CD226^+^TILs (*HR*=0.6446, 95% CI: 0.3951-1.051, *P*=0.1007, **Figure 2I**).

We also found that the frequencies of total CD8^+^TILs, CD8^+^TILs in the epithelial cell region, and CD8^+^TILs in stromal cell region were positively associated with tumor size (*P*=0.025, *P*=0.022, *P*=0.041, respectively, **Table 1**), and the frequency of CD8^+^TILs in the epithelial cell region was significantly associated with metastasis stage (M stage) (*P*=0.021, **Table 1**), and the frequency of total CD8^+^TILs could serve as an independent prognostic predictor for GC patients (HR=4.755, 95%CI:1.342-16.85, *P*=0.016, **Table 3**). Moreover, we also found that the frequency of total C226^+^ cells in stromal cell region was significantly associated with tumor size (*P*=0.015, **Table 4**).

### Prognostic value of CD226^+^CD8^+^TILs in human GC

**Figure 3:**
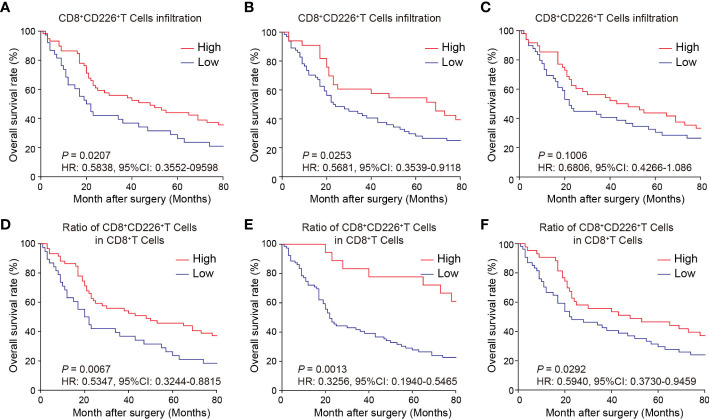
Prognostic value of CD226^+^CD8^+^TILs in human GC. **(A–C)** OS rates stratified by CD8^+^CD226^+^TIL intensities in GC tissues **(A)**. whole tissues, **(B)** epithelial cell region, **(C)** stromal cell region). **(D–F)** OS rates stratified by the ratio of CD8^+^CD226^+^TILs in CD8^+^TILs in GC tissues **(D)**. whole tissues, **(E)** epithelial cell region, **(F)** stromal cell region).

We observed here that a higher proportion of CD226^+^CD8^+^TILs in GC tissues could predict better survival of the patients. First, the GC patients with a higher proportion of CD226^+^CD8^+^TILs had better OS than those with lower number of CD226^+^CD8^+^TILs (*HR*=0.5838, 95% CI: 0.3552-0.9598, *P*=0.0207, **Figure 3A**). More specifically, the patients with higher CD226^+^CD8^+^TILs in the epithelial cell region had better OS than those with lower CD226^+^CD8^+^TILs in the same region (*HR*=0.5681, 95% CI: 0.3539-0.9118, *P*=0.0253, **Figure 3B**). The patients with higher CD226^+^CD8^+^TILs in the stromal cell region tended to have better OS than those with lower CD226^+^CD8^+^TILs in the same region (*HR*=0.6806, 95% CI: 0.4266-1.086, *P*=0.1006, **Figure 3C**). Moreover, we also evaluated the prognostic value of the frequency of CD8^+^CD226^+^TILs within total CD8^+^TILs. We found that the GC patients with a higher ratio of CD8^+^CD226^+^TILs among total CD8^+^TILs favored better OS than those with a lower ratio (HR=0.5347, 95% CI: 0.3244-0.8815, *P*=0.0067, **Figure 3D**). Besides, the GC patients with a higher ratio of CD8^+^CD226^+^TILs in total CD8^+^TILs in the epithelial cell region also favored better OS than those with a lower ratio (HR=0.3256, 95% CI: 0.1940-0.5465, *P*=0.0013, **Figure 3E**), and the similar result was found in the stromal cell region (HR=0.5940, 95% CI: 0.3730-0.9459, *P*=0.0292, **Figure 3F**).

We found that the frequencies of total CD8^+^CD226^+^TILs, and CD8^+^CD226^+^TILs in stromal cell region were significantly associated with patient’s age (*P*=0.027, *P*=0.024, respectively, **Table 5**). We also found that the frequencies of total CD8^+^CD226^+^TILs, CD8^+^CD226^+^TILs in epithelial cell region and CD8^+^CD226^+^TILs in stromal cell region were significantly associated with tumor size (*P*=0.022, *P*=0.0495, *P*=0.041, respectively, **Table 5**). We also found that the frequency of CD8^+^CD226^+^TILs in epithelial cell region was significantly and positively correlated with M stage (*P*=0.0496, **Table 5**).

Moreover, we found that the percentages of CD8^+^CD226^+^T cells/CD8^+^T cells, and CD8^+^CD226^+^T cells among CD8^+^T cells in the stromal cell region were significantly correlated with patient’s age (*P*=0.002, *P*=0.006, respectively, **Table 6**). We also found that the percentages of CD8^+^CD226^+^T cells among CD8^+^T cells and CD8^+^CD226^+^T cells among CD8^+^T cells in the stromal cell region were significantly associated with tumor size (*P*=0.022, *P*=0.023, respectively, **Table 6**). We also found that the percentages of CD8^+^CD226^+^T cells within CD8^+^T cells in the epithelial cell region and CD8^+^CD226^+^T cells within CD8^+^T cells in the stromal cell region were significantly associated with tumor stage (T stage) (*P*=0.013, *P*=0.047, respectively, **Table 6**).

**Table 6:** Uni-variate and multi-variate analysis of clinical parameters of GC patients.

Clinical parameters	Uni-variate		Multi-variate	
	*HR (95% CI)*	*P* value	*HR (95% CI)*	*P* value
Gender	1.050 (0.650-1.696)	0.841	0.977 (0.531-1.798)	0.941
Age (≥66) / (<66)	1.749 (1.085-2.818)	**0.022**	1.943 (1.031-3.661)	**0.040**
Tumor size (≥5.5) /(<5.5)	1.764 (1.100-2.828)	**0.018**	3.956 (1.415-9.140)	**0.007**
Pathological stage ((III +IV)/(I+II))	2.435 (1.236-4.796)	**0.010**	0.954 (0.529-1.722)	0.877
TNM stage ((III +IV)/(I+II))	2.504 (1.511-4.149)	**0.000**	2.829 (1.509-5.301)	**0.001**
Frequency of infiltrating PD-L1^+^Cells in Total (high/low)	1.501 (0.931-2.419)	0.095	1.001 (0.376-2.671)	0.997
Frequency of infiltrating PD-L1^+^CK^+^Cells (high/low)	2.134 (1.319-3.452)	**0.002**	1.191 (0.499-2.838)	0.693
Frequency of infiltrating PD-L1^+^Cells in SA Region (high/low)	1.606 (0.927-2.782)	0.091	1.661 (0.728-3.788)	0.228
Frequency of infiltrating CD226^+^Cells in Total (high/low)	0.554 (0.343-0.894)	**0.016**	0.492 (0.195-1.244)	0.134
Frequency of infiltrating CD226^+^Cells in EP Region (high/low)	0.528 (0.305-0.914)	**0.023**	1.018 (0.462-2.245)	0.964
Frequency of infiltrating CD226^+^Cells in SA Region (high/low)	0.641 (0.374-1.098)	0.105	1.295 (0.530-3.165)	0.571
Frequency of infiltrating CD8^+^Cells in Total (high/low)	0.868 (0.541-1.393)	0.557	4.755 (1.342-16.85)	**0.016**
Frequency of infiltrating CD8^+^Cells in EP Region (high/low)	0.777 (0.480-1.258)	0.305	0.319 (0.094-1.086)	0.067
Frequency of infiltrating CD8^+^Cells in SA Region (high/low)	0.959 (0.597-1.538)	0.861	0.826 (0.329-2.071)	0.683
Frequency of infiltrating CD8^+^CD226^+^Cells in Total (high/low)	0.563 (0.349-0.907)	**0.018**	0.612 (0.203-1.849)	0.384
Frequency of infiltrating CD8^+^CD226^+^Cells in EP Region (high/low)	0.544 (0.323-0.917)	**0.022**	1.035 (0.436-2.457)	0.937
Frequency of infiltrating CD8^+^CD226^+^Cells in SA Region (high/low)	0.669 (0.416-1.075)	0.096	0.888 (0.270-2.921)	0.844

Bold signifies P<0.05.

### Prognostic value of CD226^+^CD8^+^IFN-γ^+^TILs in human GC

**Figure 4:**
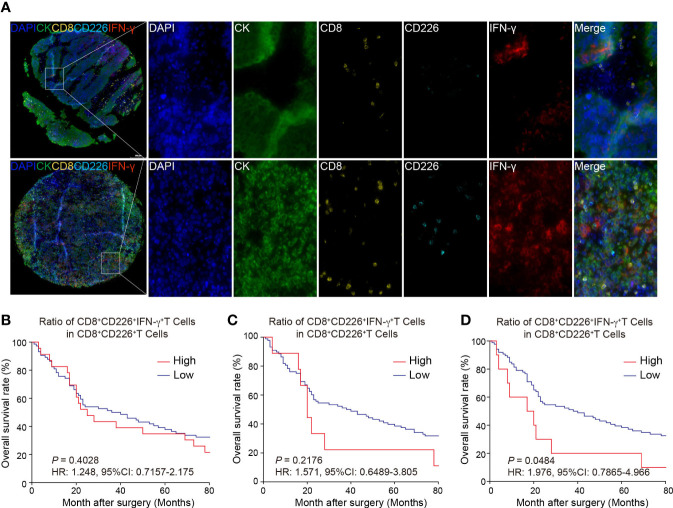
Multi-colored immunostaining of CD8, CD226, IFN-γ, and the prognostic value of CD226^+^CD8^+^IFN-γ^+^TILs in human GC. **(A)**. Representative images for staining of CD226^+^CD8^+^IFN-γ^+^TILs in GC tissues and normal tissues. **(B–D)**. OS rates stratified by the ratio of CD8^+^CD226^+^IFN-γ^+^TILs in CD8^+^CD226^+^TILs in GC tissues **(B)**. whole tissues, **(C)**. epithelial cell region, **(D)**. stromal cell region).

**Figure 4A** shows that the GC TMA section was stained with CD8 (yellow), CD226 (indigo), IFN-γ (red), and CK (green). We then evaluated the prognostic value of CD226^+^CD8^+^IFN-γ^+^TILs in human GC tissues. Generally, we did not find a significant prognostic value of the ratio of CD226^+^CD8^+^IFN-γ^+^TILs in CD8^+^CD226^+^TILs in whole tissue spot (HR=1.248, 95% CI: 0.7157-2.175, *P*=0.4028, **Figure 4B**) and also in the epithelial region (HR=1.571, 95% CI: 0.6489-3.805, *P*=0.2176, **Figure 4C**). Interestingly, we found that the GC patients with a higher ratio of CD226^+^CD8^+^IFN-γ^+^TILs in CD8^+^CD226^+^TILs in the stromal region had poorer OS than those with a lower ratio (HR=1.976, 95% CI: 0.7865-4.966, *P*=0.0484, **Figure 4D**). We found that the percentage of CD226^+^CD8^+^IFN-γ^+^T cells among CD8^+^CD226^+^T cells in the stromal cell region was significantly associated with patient’s pathological stage (*P*=0.006, **Table 2**).

### IFN-γ inhibits CD226 expression and promotes CD8^+^TIL exhaustion

**Figure 5:**
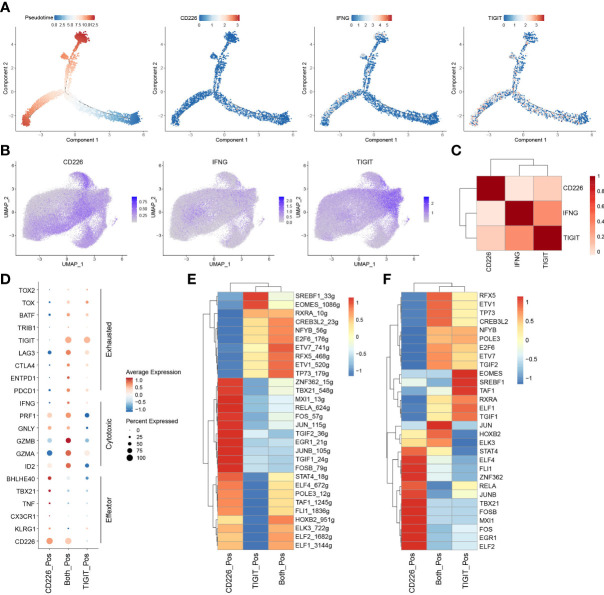
IFN-γ inhibits the expression of CD226 and promotes T-cell exhaustion. **(A)** Pseudotime plot of CD8^+^TILs in ESCA constructed by R package Monocle2. **(B)** UMAP showed imputed expressions of *CD226*, *IFNG*, and *TIGIT* in all CD8^+^TILs. **(C)** Heatmap showed the correlation among *CD226*, *IFNG*, and *TIGIT* in all CD8^+^TILs. **(D)** Dot plot showed the expressions of selected genes in CD226^+^, TIGIT^+^, and CD226^+^TIGIT^+^CD8^+^TILs. **(E)** Heatmap showed regulon score of selected transcription factors in CD226^+^, TIGIT^+^, and CD226^+^TIGIT^+^CD8^+^TILs. **(F)** Heatmap showed the expressions of transcription factors (shown in **E**) in CD226^+^, TIGIT^+^, and CD226^+^TIGIT^+^CD8^+^TILs.

To examine the biological roles of *CD226*, *IFNG*, and *TIGIT* in CD8^+^TILs, we constructed the trajectory of CD8^+^TILs in ESCA using the R package monocle2 (23). Trajectory analysis showed that *CD226* was expressed in the early developmental stage of immune cells and associated with the function of early-life T cells. *TIGIT* was expressed in the late developmental stage of immune cells, characterized by T cell exhaustion. Moreover, we surprisingly found that *IFNG* was expressed in cells with high *TIGIT* expression, indicating that IFN-γ^+^CD8^+^TILs might share the phenotype of exhausted CD8^+^TILs in TME (**Figure 5A**). To better understand the relationship between *CD226*, *IFNG*, and *TIGIT*, the imputation method by R package Magic was used to reveal their expression levels with scRNA-seq (24). The data showed that CD226^+^CD8^+^TILs had a lower expression of *IFNG* compared with TIGIT^+^CD8^+^TILs (**Figure 5B**). The correlation between *IFNG* and *TIGIT* was significantly higher than that between *IFNG* and *CD226* (**Figure 5C**). To investigate the roles of CD226 and TIGIT in CD8^+^TILs, we divided total CD8^+^TILs into three groups: CD226^+^CD8^+^, TIGIT^+^CD8^+^, and CD226^+^TIGIT^+^CD8^+^TILs. T cell activation molecule *KLRG1*, chemokine receptor *CX3CR1*, effector molecule *TNF*, and T cell activation-related transcription factors *TBX21* and *BHLHE40* were significantly up-regulated in CD226^+^CD8^+^T cells. The expressions of genes coding perforin and granzymes, such as *PRF1*, *GZMB*, and *GZMA*, were significantly increased in CD226^+^TIGIT^+^TILs and were likely to mediate cytotoxic function. The checkpoint genes *PDCD1*, *HAVCR2*, *LAG3*, and *TIGIT* were up-regulated in CD226^+^TIGIT^+^CD8^+^ and TIGIT^+^CD8^+^TILs, contributing to T cell dysfunction and exhaustion (**Figure 5D**). Therefore, we proposed that IFN-γ might inhibit the expression of co-stimulatory molecules, such as CD226, in CD8^+^TILs and then promote T-cell exhaustion by affecting a subset of transcription factors associated with exhaustion. The transcriptional regulatory network of CD8^+^T cells was created by R package SCENIC among three populations (25). Results showed that the AP-1 family (*JUN*, *JUNB*, *JUND*, *FOS*, and *FOSB*) and ETS family TF (*ELF1*, *ELF2*, *ELF4*, *ELK3*, and *FIL1*) were highly expressed in CD226^+^CD8^+^TIL cells with increased transcriptional regulation activity (**Figures 5E**, **F**). These data suggested that CD226 played a critical role in amplifying TCR signaling.

### Correlation between *CD226* and specific cell components in the TME of GC

**Figure 6:**
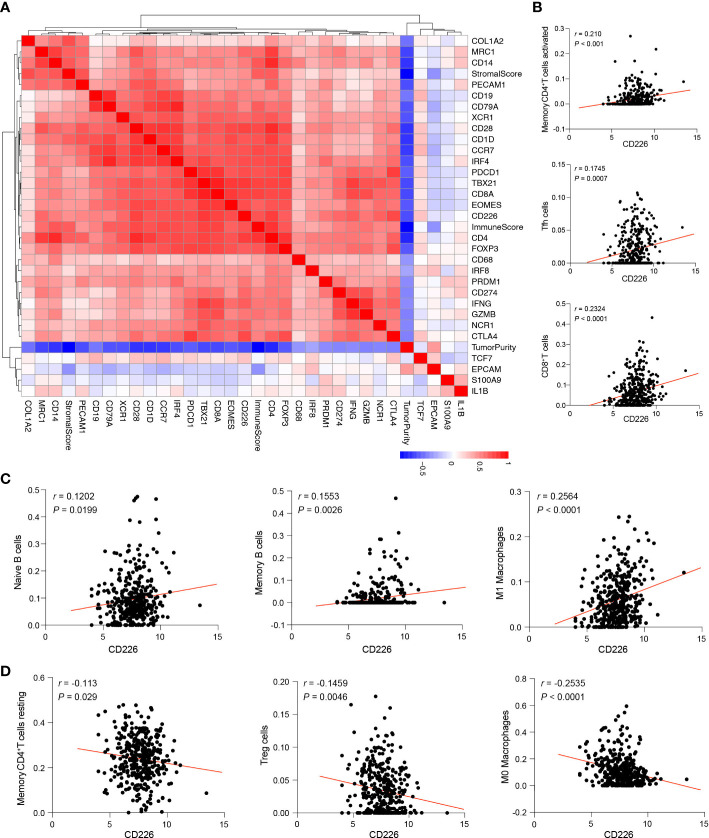
Correlation between the expression of *CD226* and other cellular components in the TME. **(A)**. Heatmap showed the correlation of *CD226* and tumor purity, stromal score, and immune score, as well as with immune-related genes in TCGA STAD. **(B–D)**. The dot plot showed the correlation between CD226 and cellular components scored by CIBERSORT and gene sets LM22.

RNA-seq data from TCGA STAD were downloaded, and the relationship between *CD226* and tumor purity, stromal and immune score, as well as with signature genes of the specific cells and T cell activation effectors in TME, were analyzed (**Figure 6A**). *CD226* was negatively correlated with tumor purity and positively correlated with the immune score. *CD226* was significantly enriched in the module of effector T cells. These T-cell enriched modules included genes encoding effector molecules (**Figure 6A**). CIBERSORT was used to produce the immune scores of 22 immune cell subsets (26). We found that *CD226* was positively correlated with memory CD4^+^T cells, T follicular helper cells (Tfh), and CD8^+^T cells (*P*<0.001, **Figure 6B**). Furthermore, we found a positive correlation between *CD226* and naïve B cells, memory B cells, and tumor-associated type I macrophages (M1) (**Figure 6C**). *CD226* was significantly and negatively associated with resting memory CD4^+^T cells, Tregs, and M0 macrophages, the populations of suppressive characteristics (*P*<0.05, **Figure 6D**). Collectively, *CD226* was not only regulated by the activation of immune cells but also inhibited by immunosuppressive cells of TME.

## Discussion

As crucial immune checkpoint molecules, T-cell inhibitory receptors, including CTLA-4, PD-1, TIM-3, LAG-3, and TIGIT, are essential in limiting immunopathology and terminating effective immune response, while they can also inhibit effective anti-tumor immunity (27). In recent decades, the immunotherapeutic antibodies against these inhibitory receptors have been designed to enhance and revitalize tumor antigen-specific T-cell response, causing definite or promising therapeutic effects in cancer patients (28). Currently, several clinical trials using immune checkpoint blockade (ICB) against GC, such as ATTRACTION-2, KEYNOTE-059, AIOSTO-0417, NCT03647969, and NCT02872116, have shown anti-tumor activity and manageable toxicity (29). However, it still remains largely uncertain when exploring the genomic and molecular biomarkers of response and resistance to ICB in the context of the complex TME of GC. Zeng et al. have established the TME score, providing a valuable predictor for precision immunotherapy against GC (30, 31).

It has been demonstrated that as a critical activated receptor on CD8^+^T cells, the expression of CD226 and the frequency of CD226^+^CD8^+^TILs are significantly and positively associated with the clinical benefit of ICB in certain human cancers (17, 32). Several reports have also shown that CD226 signaling in CD8^+^T cells plays a vital role in the anti-tumor response in the mouse tumor model (13, 32, 33). First, CD226-neutralizing monoclonal antibodies can eliminate the combined effect of anti-PD-L1 and anti-TIGIT antibodies (33). In addition, CD8^+^T cells with reduced or missing expression of CD226 show dysfunction and are related to drug resistance of tumor immunotherapy (13). Moreover, the blockade of TIGIT and PD-1 can help restore the CD226 signaling on CD8^+^TILs and optimize the CD8^+^T cell-mediated anti-tumor response (32). In our present study, we analyzed various phenotypes of T cells expressing CD226, CD8, and IFN-γ in tissue samples from GC patients. Higher expression of CD226 was associated with better OS of the patients. Similar results were also found among patients with a higher frequency of CD226^+^CD8^+^TILs, suggesting that CD226 played a vital role in maintaining immune surveillance in the TME of GC.

Also shown in **Table 3**, we have emphasized that CD8^+^TILs, CD226^+^CD8^+^TILs, and CD226^+^CD8^+^IFN-γ^+^TILs could be prognostic predictors for GC patients. However, their functions as independent prognostic indicators were not consistent. Our data also showed that the higher frequency of IFN-γ^+^CD8^+^CD226^+^TILs in the GC tissues indicated poorer OS of the GC patients. Furthermore, scRNA-seq and TCGA data revealed that *CD226* was down-regulated, while the expression of *TIGIT* was increased in TME. By an integrative analysis of the single-cell transcriptome and the T cell receptor repertoire, we analyzed the expressions of *CD226*, *TIGIT*, and *IFNG* in CD8^+^TILs. CD226^+^CD8^+^TILs expressed transcripts encoding effector molecules and regulatory proteins, while TIGIT^+^CD8^+^TILs were enriched with genes leading to the T-cell exhaustion, as well as an immune checkpoint gene expression profile of *PDCD1*, *HAVCR2*, *LAG3*, and *TIGIT*. Besides, our findings indicated that CD226 contacted memory T/B cells, effector T cells, Tfh cells, and macrophages. In cancer progression, *CD226* was negatively regulated by TME-driven immune suppression, while *IFNG* and *TIGIT* were highly expressed in specific TILs. CD8^+^CD226^+^IFN-γ^+^T cell population might develop a high TIGIT expression pattern, resulting in decreased CD8^+^T cell- or NK cell-mediated tumor reactivity. Our data suggested that CD8^+^CD226^+^IFN-γ^+^T cells represented a status of immune exhaustion of CD8^+^T cells during GC progression. In TME, the higher frequency of IFN-γ^+^CD8^+^CD226^+^TILs was correlated with up-regulated TIGIT expression, which could serve as an essential and potential biomarker to predict the progress and immune evasion in GC patients.

## Data availability statement

The original contributions presented in the study are included in the article/**Supplementary Material.** Further inquiries can be directed to the corresponding authors.

## Ethics statement

The studies involving human participants were reviewed and approved by Shanghai Outdo Biotech Co., Ltd., Shanghai, China (SHYJS-CP-1507004). The patients/participants provided their written informed consent to participate in this study.

## Author contributions

(I) Conception and design: LC, XuZ, and JJ. (II) Administrative support: LC, XuZ, and JJ. (III) Provision of study materials or patients: HH, ZH, JG, JY, and JC. (IV) Collection and assembly of data: HH, ZH, BX, SW, and XiZ. (V) Data analysis and interpretation: HH, ZH, and LC. (VI) Manuscript writing: All authors. (VII) Final approval of manuscript: All authors.
