# Supplementary figures and images for: CD226 identifies functional CD8+T cells in the tumor microenvironment and predicts a better outcome for human gastric cancer
Source: Front Immunol. 2023 Mar 28;14:1150803. doi: 10.3389/fimmu.2023.1150803 (PMC10086426; doi:10.3389/fimmu.2023.1150803)

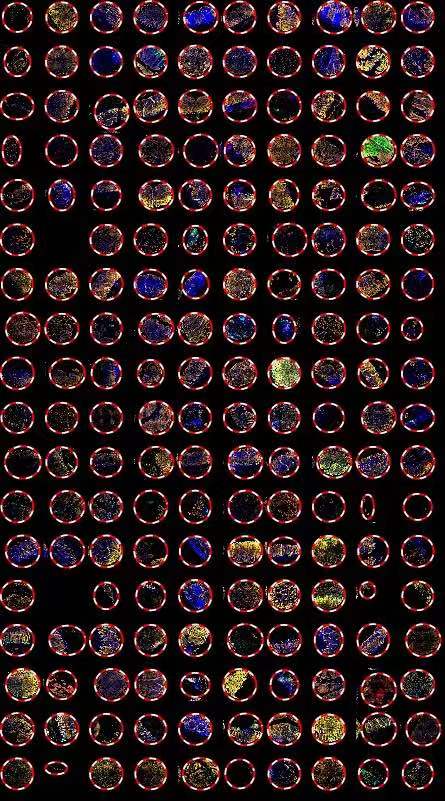

Supplement: Supplementary file 3 [file Image_1.jpeg]

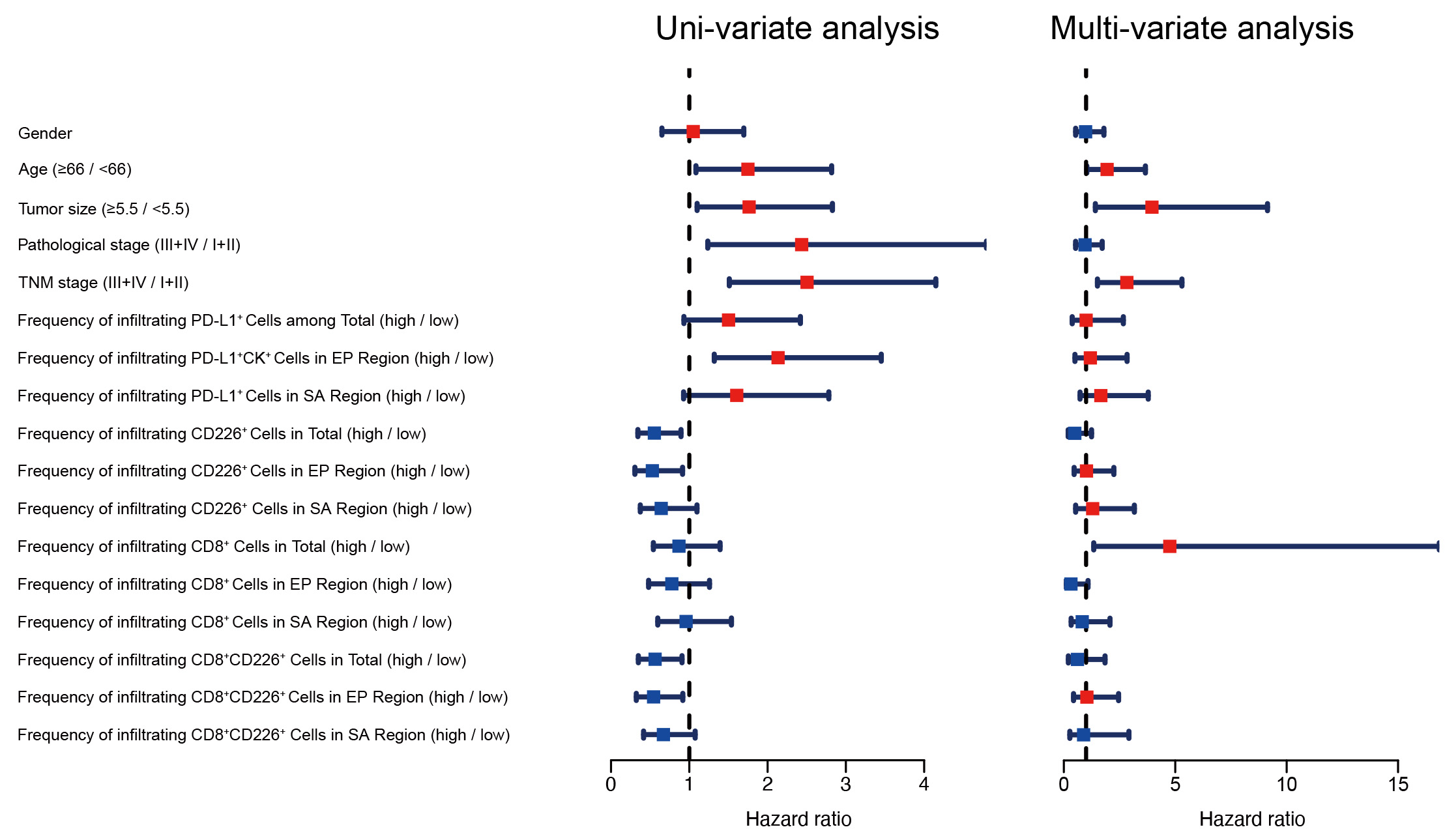

Supplement: Supplementary file 4 [file Image_2.jpeg]
